# Utilization of machine learning to test the impact of cognitive processing and emotion recognition on the development of PTSD following trauma exposure

**DOI:** 10.1186/s12888-020-02728-4

**Published:** 2020-06-23

**Authors:** Mareike Augsburger, Isaac R. Galatzer-Levy

**Affiliations:** 1 Department of Psychology, University of Zurich, Binzmuehlestrasse 14, 8050 New York, USA; 2 AI Cure, New York, USA; 3 New York University School of Medicine, New York, USA

**Keywords:** PTSD, Machine learning, Neuro-cognitive functioning, Emotion recognition, Symptom development

## Abstract

**Background:**

Though lifetime exposure to traumatic events is significant, only a minority of individuals develops symptoms of posttraumatic stress disorder (PTSD). Post-trauma alterations in neurocognitive and affective functioning are likely to reflect changes in underlying brain networks that are predictive of PTSD. These constructs are assumed to interact in a highly complex way. The aim of this exploratory study was to apply machine learning models to investigate the contribution of these interactions on PTSD symptom development and identify measures indicative of circuit related dysfunction.

**Methods:**

*N* = 94 participants admitted to the emergency room of an inner-city hospital after trauma exposure completed a battery of neurocognitive and emotional tests 1 month after the incident. Different machine learning algorithms were applied to predict PTSD symptom severity and clusters after 3 months based.

**Results:**

Overall, model accuracy did not differ between PTSD clusters, though the importance of cognitive and emotional domains demonstrated both key differences and overlap. Alterations in higher-order executive functioning, speed of information processing, and processing of emotionally incongruent cues were the most important predictors.

**Conclusions:**

Data-driven approaches are a powerful tool to investigate complex interactions and can enhance the mechanistic understanding of PTSD. The study identifies important relationships between cognitive processing and emotion recognition that may be valuable to predict and understand mechanisms of risk and resilience responses to trauma prospectively.

## Background

The majority of individuals will experience a life-threatening or potentially traumatic event across their life course that puts them at risk for post-traumatic psychopathology [1]. According to the Diagnostic and Statistical Manual of Mental Disorders (DSM-5), posttraumatic stress disorder (PTSD) is characterized by four symptom clusters: Constant re-experiencing (cluster B), avoidance of stimuli associated with the traumatic event (cluster C), increased physiological arousal (cluster E), along with negative alterations in mood and cognition (cluster D), thus resulting in a significant impairment in daily life [2]. However, proportions of those suffering from chronic PTSD symptoms are relatively small compared to the high incidence of trauma exposure (c.f [3].). Yet, the early identification of individuals at risk for later pathologic development has remained challenging [4, 5].

The relationship between cognitive and emotional information-processing of stimuli in the brain presents a hallmark characteristic of PTSD and is reflected in cognitive and affective dysregulations [6–8]. In their review, Aupperle et al. point to the importance of executive dysfunction, attention, working memory disturbances, sustained attention, inhibition, and flexibility in and switching of attention. In contrast, dimensions of planning and strategy seem to be less affected [9]. Consistently, information processing speed, verbal learning, verbal memory, attention, and working memory have demonstrated the strongest effects in differentiating individuals with PTSD from their healthy counterparts in a meta-analysis [10].

Emotional information processing has also been shown to be reduced in PTSD. More specifically, patients with PTSD consistently demonstrate deficits in recognition of correct emotions in facial stimuli compared to healthy controls [11]. Researchers argue that an increased reactivity to body sensations after trauma exposure along with increased aversion may lead to avoidance [12]. Taken together, understanding the impact and relation between dysregulations in cognitive and emotion information processing following trauma can inform prognosis, diagnosis, and treatment selection as it relates to PTSD. However, these dysregulations cannot be considered as distinct processes but might reflect overlapping constructs of underlying neural mechanisms [13, 14]. For instance, it has been shown that impaired inhibition affects the controllability of emotional cues [8, 15], and thus might lead to symptoms of re-experiencing [9]. Moreover, a study with veterans demonstrated that only interactions between emotional reactivity and impairment in executive functioning were associated with more severe PTSD symptoms, but none of the variables alone [6]. Finally. treatment response in PTSD has been shown to be associated with distinct predictors of cognitive and emotional processes [7]. This indicates that the nature of the relationship as it predicts distinct domains is necessary to identify risk and understand the underlying mechanisms.

In light of these findings combining information from cognitive and emotion processing might provide a better understanding of how PTSD develops after trauma exposure. Since studies suggest that interactions between specific facets on various stages facilitate the exacerbation of PTSD symptoms, (c.f [6, 9, 13, 15].), an analysis approach is required that can accommodate complex interactions of highly-dimensional data. Hereby, classical statistical testing methods quickly reach their limits due to problems associated with inflated error probability in light of multiple testing and reduced power. Furthermore, only a limited number of predictors can be included in traditional models at the same time. A promising approach is offered by machine learning algorithms. Such models can be utilized to determine shared predictive accuracy of a variable set and can be used to gain insights into interactions between variables. They can accommodate relatively large variable-to-sample ratios to identify interactions across multiple variables [16, 17]. For instance, unbiased predictions could be derived even with a small sample of *N* = 40 [18] and sample size does not affect model robustness when applying nested cross-validation procedures [19]. Finally, machine learning models have been increasingly applied for investigating predictors for outcomes of health-related behavior (e.g. [16, 20]), and particularly in the area of traumatic stress (e.g., [21, 22]). In light of the evidence that cognitive and emotional information processing are important in the exacerbation of PTSD, the aim of the current study was to characterize and test the relationship and predictive accuracy of multiple relevant domains of cognitive processing and emotion recognition as they impact PTSD and distinct symptom cluster severity. More specifically, the study sought to investigate the potential of different machine learning models and its predictive capabilities with respect to identify individuals with elevated PTSD symptom severity by simultaneously combining a number of variables that might have altered post-exposure. Thus, we deliberately focused on predictors from cognitive processing and emotion recognition domains irrespective of other variables that are known to serve as a risk factor for PTSD. The selection of specific variables was based on most significant associations and cognitive tests reported in the review from Aupperle et al. [9] and Scott et al. [10]. More specifically, tests associated with attention/working memory, sustained attention and inhibition, flexibility, verbal memory and processing speed were chosen. Regarding emotional processing, recognition of emotions in facial stimuli were investigated, thus following previous investigations [11]. Since this study was of exploratory nature to test the applicability of machine learning models within this setting, no further hypotheses were specified.

## Methods

This study was part of a larger research project assessing trajectories of mental health after exposure to a traumatic event (NYU/Bellevue Stress and Resilience longitudinal study).

### Participants

English-speaking adults between 18 and 70 who were admitted to the General Emergency Department (ED) of the Bellevue Hospital Center, New York City after exposure to a potentially traumatic event were asked for study participation. An event was considered traumatic as defined in the diagnostic classification for PTSD in DSM-IV (criterion A) [23]. Cases of domestic violence were not included. Further inclusion criteria were: no symptoms of past or present psychosis; no admission to the Psychiatric ED; and not currently being in custody of the police or the Department of Corrections. In order to increase risk for pathologic development following traumatic experiences, at the initial screening in the ED individuals were asked about their current level of a distress on a Subjective Units of Distress Scale (SUDS) ranging 0–100. A score ≥ 60 or an intense emotional reaction during the interview was considered eligible for study participation.

Out of all persons admitted to the ED due to a traumatic event, *n* = 338 individuals were eligible for study participation. Reasons for admission were falls (21%), bike accidents (16%), hits as a pedestrian (19%), motor-vehicle accidents (17%), assaults (10%) and other event types (17%) such as gun-shots, lacerations or seizures. *N* = 111 individuals took part in the 1-month follow-up, and *n* = 105 completed 3-month follow-up, respectively. In the current analyses, all participants with complete neuro-cognitive and emotional assessment were included (*N* = 94). Of these, *n* = 58 were men and *n* = 36 were women. Mean age of the final sample was 36.95 years (SD = 13.83, 19–67 years) at baseline and participants had in average 14.99 years of education (SD = 3.35, 4–18). The majority (50%) were Caucasians, followed by African Americans (19%), Asians or Hispanics (both 3%). 9% preferred not to specify, and 16% indicated another other ethnic group. There was no significant difference between those having completed the assessment and non-completers regarding initial SUDS rating, age, ethnic group, level of education. However, completers reported significantly more bike accidents and fewer assaults than expected (both *p* < .05).

### Procedures

All new admissions to the hospital after trauma exposure were checked for study eligibility, starting in fall 2014. If eligible and after having provided informed consent, participants were initially assessed within the emergency room setting and followed-up within the first week after discharge from the hospital for a phone screening of 30-min mean duration (not further reported here).

Participants were again invited after 1-month for an in-visit. After 3-months post-incident a follow-up appointment was scheduled, lasting about 20 min. The current analyses include this 1 and 3 months follow-up information. Data collection for later follow-ups was still in progress. For the 1-month assessment, participants got reimbursed with $100, and $30 for the 3-months follow-up, respectively.

The research team was composed of an experienced research coordinator and several research assistants (Master or PhD students) working under close supervision. They had received intense training in handling trauma populations.

### Measures

#### Predictors at 1-month follow-up: Neuro-cognitive functioning and emotion recognition

The computer-assisted and widely applied test battery “WebNeuro” provides neuropsychological tests for cognitive performance. Conformity to touch-screen equivalent (IntegNeuro) has been demonstrated [24], and the latter presents comparable validity and reliability in comparison to paper-and-pencil test versions [25, 26]. Relevant constructs and tests were selected according to the previously reported findings with the exception of the Choice Reaction Time Test. Outcome measures within each test were selected according to the WebNeuro manual [27]. In addition, variables with high collinearity (> .80) were removed by inspecting pair-wise correlations and removing the variable with largest mean absolute correlation. The labeling of constructs for tests also follows the WebNeuro manual [27] and can differ from classifications of domains used by other authors.

*Speed of information processing* was measured with the *Choice Reaction Time* test. Participants had to identify the correct position of a green illuminated target appearing at one of four target positions (black-filled circles) by pressing the matching button as quickly as possible. In total, there were 20 trials and targets appeared in a pseudo-random order at one of the four positions. Reaction time (RT) was used. This test was chosen because it is less affected by mild traumatic brain injury [28].

##### Sustained attention

In the *Continuous Performance Task,* a series with one of four letters (D, B, G, or C) was presented. Participants pressed a button when two identical letters consecutively appeared. In total, 125 letters were presented (85 non-target and 20 target letters). Errors of commission (false identification of non-targets), errors of omission (non-identification of targets) and RT were used.

##### Attentional flexibility

Similar to the *Trail Making Test* Version B [29], 13 digits (1–13) and 12 letters (A-L) were presented. Participants were asked to touch digits and letters in an alternating and ascending sequence (1A2B, …). Time to completion was used.

##### Executive functioning/inhibition

These domains were measured with a *Go/No-Go* and a *Verbal Interference Task.* For the *Go/No-Go* task participants were asked to hit the space bar as quickly as possible if the word “*press*” was shown in green letters and inhibit the movement accordingly, if the word was shown in red letters. Errors of commission, errors of omission and RT were chosen as relevant variables. The *Verbal Inference Task* is similar to the Word-Color-Stroop task [30]. Participants were asked to identify name and color of words presented with congruent or incongruent color-word combinations. Errors during incongruent trials was used.

##### Attention and working memory

In the *Digit Span* test, a series of digits, gradually increasing from 3 to 9 digits, was recalled. Maximum recall span was measured.

##### Verbal learning capacity

Equivalent to the California Verbal Learning Test, a list comprised of 12 words was presented in 3 consecutive trials. Participants were asked for immediate recall after each trial. Mean number of correctly recalled words was chosen.

##### Implicit emotion recognition

The standardized stimuli set [31] includes faces of 12 persons (both 6 women and men) with a total of 72 facial expressions. In the first part of the task (*explicit emotion recognition*), a pseudorandom order of 48 faces from 8 different persons was presented. Participants were asked to select emotion labels corresponding to six facial expressions (happiness, fear, sadness, anger, disgust, and neutral). After a series of filter tasks of about 20 min, the *implicit emotion recognition* task was applied. A random selection of 24 facial expressions with six emotions (two male and two female sets of emotions each) from the first task were presented in a pseudo-random order together with 24 completely new stimuli with otherwise identical properties. In each trial, participants had to select the previously presented face.

Since, the study aim was to measure an emotional bias by influence of a previous exposition towards emotions on later emotion recognition capabilities and thus the tendency to avoid particular emotions automatically, only the implicit emotion recognition task was included in the analyses. Due to negligible differences in recognition of specific emotions, scores were averaged across all emotions. Accuracy for both incongruent (different primer and distracting emotions) trials and congruent (same primer and distracting emotions) trials was used as variables. For further information about the task, see [32].

#### Outcome at 3-months: PTSD overall symptom severity and cluster-specific symptoms

The Posttraumatic Symptom Checklist for DSM-5 (PCL-5) was used [33]. It is comprised of 20 items and each corresponds to a DSM-5 diagnostic criterion for PTSD. Participants indicate the severity of symptoms during the past month on a 5-point Likert scale ranging from 0 (*not at all*) to 4 (*extremely*), resulting in total sum score range from 0 to 80 reflecting overall symptom severity. Additionally, severity of diagnostic clusters of PTSD can be computed. Intrusion symptoms (cluster B) can range from 0 to 20, symptoms of avoidance (cluster C) between 0 and 8, negative alterations in cognition and mood (cluster D) between 0 and 28, and alterations in arousal and activity (cluster E) between 0 and 24, respectively. The PCL-5 shows excellent psychometric properties and is one of the most-used self-report measures for PTSD [34]. A cut-off value > 33 was considered indicative of a provisional diagnosis for PTSD. For the analysis, sum scores were used to be able to include as much information as possible for learning associations. Cronbach alpha was .95 in the current sample for the total sum score.

### Data analysis

#### Missing values and data pre-processing

In total, 10% of the dataset had missing values. These were imputed using a recursive partitioning approach by means of random forest, which is suitable for mixed-data. Due to its non-parametric fashion, random forests do not require a-priori specification of variable distributions. The algorithm outperforms other common implementation techniques in terms of imputation error such as *k*-nearest neighbor or multiple imputation by chained equations [35, 36]. Regarding data pre-processing, variables were scaled and centered if required for a specific algorithm (e.g. for Support Vector Machines or Neural Networks).

In order to explore dysfunctions, all test scores were compared to a normative cohort using peer regression modeling for age, gender and education [27]. For emotion recognition, only norms for emotion identification were reported. Z-scores within 1SD were considered average, and ≤ − 1 but ≥ − 2 were considered borderline below average performance [37].

### Machine learning algorithms

The implementation followed recommended procedures (see [38]). Since predictive performance within a given dataset is unknown in advance, it is recommended to test a range of models (c.f [38].). In the current studies, supervised algorithms that have been frequently applied in mental health studies were compared. More specifically, support vector machines (SVMs), random forests, boosted models and neural networks were tested (c.f [39, 40].). In addition, two other models (basic decision trees and bagged trees) were applied that have been shown to be robust towards noisy data and applicable in a broad range of settings whilst being somewhat interpretable (c.f [38].).

SVMs are characterized by finding a linear separation (hyperplane) that best differentiates the outcome based on the predictor values [41]. Furthermore, basic classification and regression trees (CART), random forests (RF), boosted and bagged models all belong to the category of decision trees. In these models, the outcome is predicted by partitioning each predictor based on a series of if-then statements. During model bagging several decision trees are averaged by repeated resampling of the data [42]. Furthermore, RF is also a tree-based ensemble learning technique. It works similar to bagged trees, but at each step of the tree-building procedure only random subset of the predictors is included (see [43]). Boosted regression trees are also a tree-ensemble method but in contrast to other techniques, each step during model building is based on the residuals that could not be explained in the step before (see [44]). Finally, neural networks combine predictors into multiple hidden units. In a second step, the outcome is modeled by these hidden units (see [45]). Model specification details are reported in the online supplementary material.

As stated above, machine learning models are characterized by model-inherent parameters that are systematically tuned to receive best prediction performance. In order to avoid over-fitting (models perform well in the current sample but can be poorly generalized), more complex models are penalized during the model building process. Furthermore, at each step during tuning, only a small portion of data is used. A left-out set is subsequently used for evaluating predictive performance. In the current study, 10-fold cross-validation with five repetitions was applied with model building at each step of the cross-validation process. This nested procedure was chosen instead of a separate training and test set in light of the limited sample size [38]. It has shown to result in robust estimates in small samples [18, 19].

In order to evaluate predictive performance, two indices were used for quantifying prediction error. Root means squared error (RMSE) indicates the magnitude of residuals left in the model derived from observed minus predicted values [38]. Thus, lower values of RMSE were preferred. Since this measure is scale-depended, it cannot be used to compare model performance across different outcomes. For this reason, R-squared (squared observed versus fitted values) was used. R-squared is interpreted as the proportion of variation in the outcome that can be explained by the predictors. Thus, higher values were preferred. Both RMSE and R-squared are recommended when testing the predictive capability of machine learning models with continuous outcome [38]. Indices derived from each step were averaged to derive one single final estimate. Since there is no recommended cutoff for optimal values of RMSE and R-squared, differences in model performance were compared based on pairwise t-tests with Bonferroni adjustment for multiple testing. In addition, variable importance scores were computed. Values were scaled from 0 to 100 with larger values indicating higher contribution in the model. Whilst no statistical test is available for drawing conclusions about the relevance of specific predictors, we chose to explore patterns. For this reason, the three most important predictors for each model were considered. Finally, in order to quantify interactions between features, Friedman’s H was calculated (see [46]). Friedman’s H can be interpreted as the portion of variance that is explained by the interaction when controlling for other effects. The index can take values between 0 and 1. Currently there is no statistical significance test available so again we described patterns of the five most frequently occurring two-way interactions across models.

*R* [47] with packages *missForest* [48] and *caret* [49] as well as respective dependencies were used for statistical analyses.

## Results

### Descriptive statistics

Table 1 displays descriptive statistics of all predictor variables at the 1-month assessment. A comparison with the normative cohort revealed only minor deviations (z-scores < |.5|). Only in the continuous performance test, participants’ mean reaction time was in a borderline range (for details see Table 1A in the online supplementary material). Thus, there is no evidence for severe neurocognitive impairment in this sample.

**Table 1:** Mean and standard deviation (SD) of predictor variables

Test	Variable [unit]	Mean (SD)
**Choice Reaction Time**	RT [ms]	451.73 (177.66)
**Continuous Performance**	# of Errors	7.49 (18.19)
	RT [ms]	593.05 (122.55)
**Go/No-Go**	RT [ms]	321.64 (64.04)
	# of Errors	6.00 (6.03)
**Verbal Interference**	# of Errors	1.85 (3.91)
**Digit span**	# of Maximum Digits	6.52 (1.77)
**Verbal learning**	# of Errors	4.46 (7.61)
**Digit-Letter-test**	Completion Time [ms]	62,241.62 (54,964.60)
	# of Errors	1.80 (3.78)
**Face recognition**	Accuracy Incongruent [%]	85.61% (19.90)
	Accuracy Congruent [%]	95.04% (9.16)

*RT* Reaction Time, *ms* milliseconds, # number

Regarding the outcome, mean PTSD symptom severity as measured by the PCL-5 was 23.38 (SD = 15.74), range 0–62. Of these, 24% met the cut-off above33 indicative of a provisional diagnosis. For disorder-specific sub-clusters, mean scores were 5.11 (SD = 4.12) for cluster intrusion symptoms, 2.68 (SD = 2.10) for avoidance, 8.29 (SD = 6.29) for changes in mood and cognition, and 7.45 (SD = 5.04) for hyperarousal, respectively.

### Data-driven predictions within outcomes

Final model parameters and associated fit values are reported in Tables 2A-6A in the online supportive material. Comparing accuracy within the same outcome for overall PTSD symptom severity, values for RMSE were between 14.32 (boosted tress) and 15.53 (CART model). Pairwise t-tests indicated that SVM and CART models were significantly worse than the bagged tree and random forest model (all *p* < .001). In addition, the random forest model was also superior to the neural network model (*p* = .04). No other significant differences emerged (all *p* > .05). Thus, the random forest model was considered the optimal model for overall PTSD scores (see Figure S1 in the online supportive material for details).

For symptoms of re-experiencing (PTSD cluster B), RMSE values of the tree-based ensemble methods (bagged tree, boosted tree and random forest; RMSE between 3.53–3.58) were significantly lower compared to the SVM (RMSE = 3.87, all *p* < .004) and CART models (RMSE = 3.88, all *p* < .05), but not to the neural network model (RMSE = 3.82, *p* = .51 for bagged trees and *p* = .18 for random forests). The boosted tree model came close to significance (*p* = .08) and was therefore chosen as best model (see Figure S2 in the online supportive material for details).

Concerning symptoms of avoidance (PTSD cluster C), random forest (RMSE = 1.92) and boosted tree (RMSE = 1.958) models had significantly lower values than the SVM (RMSE = 3.87, both *p* < .001), CART (RMSE = 2.03, both *p* < .04) and the neural network model (RMSE = 2.11, both *p* < .009). Random forest and boosted models did not differ from each other (*p* = .46) and the bagged tree model (RMSE = 1.963, both *p* > .5). Yet, the bagged tree model was only superior to the SVM model (*p* <. 001), but not the CART and neural network model (*p* = > .17). Consequently, random forest and boosted tree models were chosen as final models for PTSD cluster C (see Figure S3 in the online supportive material for details).

For alterations in cognition and mood (PTSD cluster D), a very similar pattern occurred. Both the random forest (RMSE = 5.82) and the boosted tree (RMSE = 5.81) model were significantly lower than the SVM (RMSE = 6.08), CART (RMSE = 6.46) and neural network (RMSE = 6.63) model (all *p* < .05) but did not differ from the bagged tree model (RMSE = 5.88, *p* > .9). The bagged tree model was superior to the CART and neural network (both *p* < .001), but not the SVM (*p* = .06) model. Again, random forest and boosted models were considered optimal for PTSD cluster D (see Figure S4 in the online supportive material for details).

For symptoms of hyper-arousal (PTSD cluster E), the two models with lowest scores, that is the CART (RMSE = 4.64) and boosted tree model (RMSE = 4.62), were significantly better than the SVM (RMSE = 4.99, both *p* < .001) and neural network (RMSE = 5.10, both *p* < .05) models. Only the boosted tree model was also superior to the bagged tree (RMSE = 4.76, *p* = .03) and close to significance for the random forest model (RMSE = 4.75, *p* = .08). Accordingly, it was chosen as the optimal model for the prediction of PTSD cluster E (see Figure S5 in the online supportive material for details).

### Model performance across outcomes

**Fig. 1:**
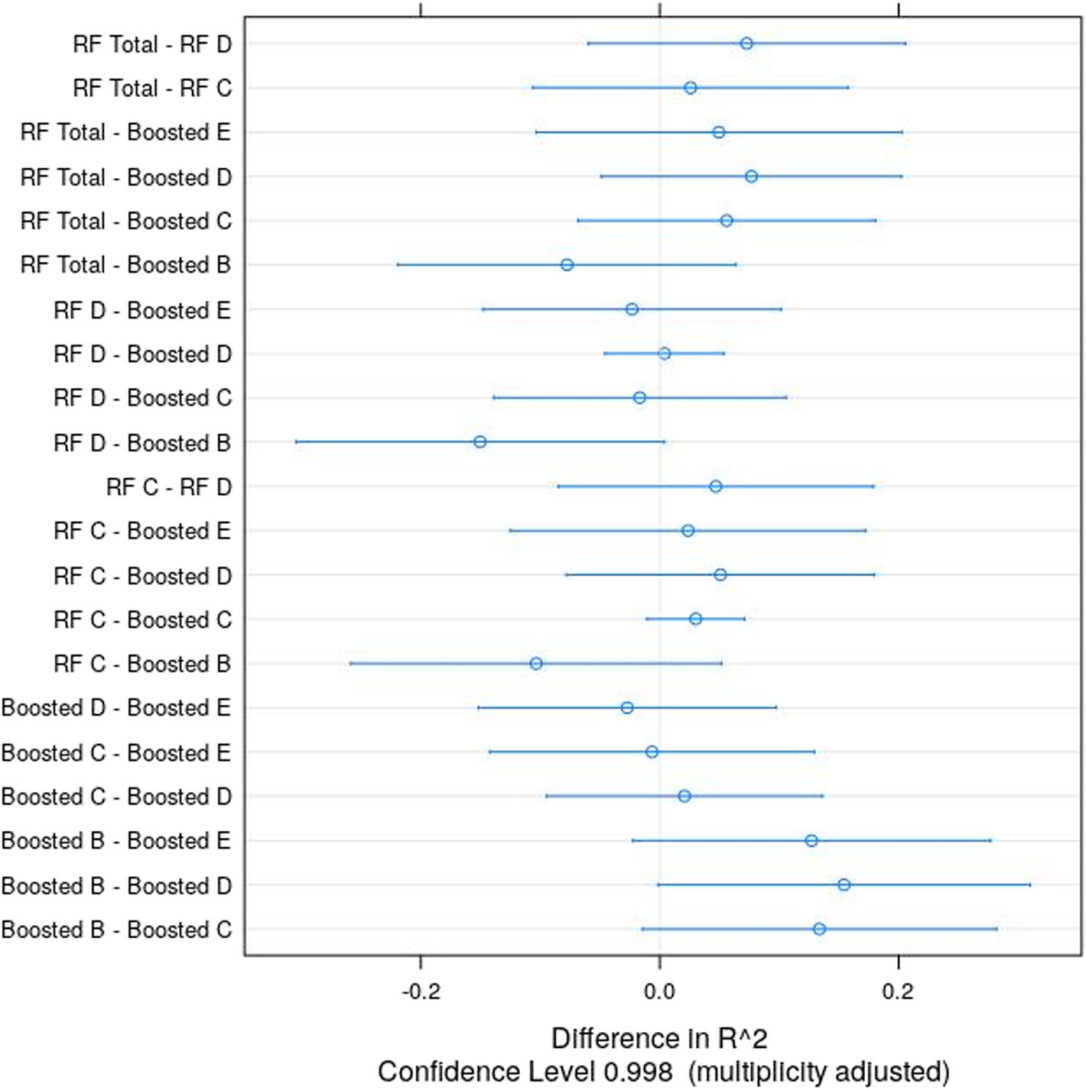
Differences in mean R-squared and associated confidence intervals for pairwise comparisons of random forest (RF) and boosted models. The letters refer to the PTSD cluster B-E, “Total” refers to the model with overall symptom severity

In a next step, best performing models per outcome were compared based on maximized R-squared, That is, the random forest model was chosen for overall PTSD symptom severity (Rsquared = .28), cluster C (Rsquared = .25) and cluster D (Rsquared = .20). Furthermore, boosted models were selected for PTSD cluster B (Rsquared = .36), cluster C (Rsquared = .22), cluster D (Rsquared = .20) and cluster E (Rsquared = .23). Despite these descriptive differences, no model performed exclusively better (all *p* > .19). There was only a non-significant trend for the boosted model for cluster B outperforming the random forest model for cluster C (*p* = .06) as well as the boosted model for cluster D (*p* = .05). See Fig. 1 for details.

### Importance of predictor variables

**Fig. 2:**
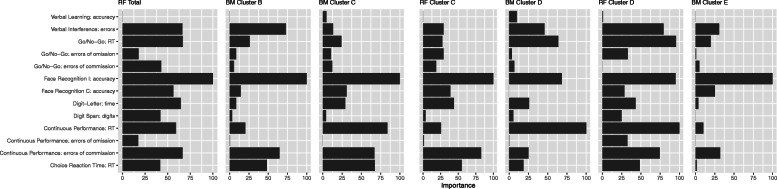
Variable Importance of predictors for all final models scaled from 0 to 100

Figure 2 visualizes variable importance for all selected models. Of note, some variables had almost equal scores and were therefore all considered, thus including four predictors for overall symptom severity and the boosted model for cluster C.

Accuracy during incongruent trials in the *Face Recognition* task was the most or second most important predictor in all models. This was followed by errors in the *Verbal Interference* task for PTSD symptom severity (together with RT in the *Go/No-Go* task), cluster B and E, and errors of commission in the *Continuous Performance* test for overall PTSD, cluster B, C (together with RT in the *Choice Reaction Time* test*)* and E. Furthermore, regarding the prediction of cluster D, RTs in the *Go/No-Go* and *Continuous Performance* tests were relevant. The latter test was also the second most important predictor for the boosted cluster C model.

In addition, accuracy in the *Verbal Learning* memory task (for overall PTSD symptoms, boosted model cluster B and E as well as the random forest model cluster C), errors of commission in the *Go/No-Go* task (random forest model for cluster D), number of digits in the *Digit Span* test and errors of omission in the *Continuous Performance* test (boosted model for cluster E) all had zero importance.

### Interactions

**Fig. 3:**
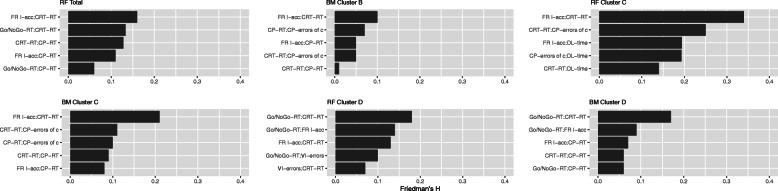
Friedman’s H values for the most important 2-way interactions for all models. For boosted model cluster E, no interaction terms were included in the final model (tree depth = 1, see Table 4A in the online supportive material). RT = reaction time, FR I-acc = Face recognition accuracy during incongruent trials; CRT = Choice Reaction Time, CP = Continuous Performance, errors of c = errors of commission, VI = verbal interference, DL = Digit-Letter

Figure 3 plots the five most important two-way interactions for each model based on Friedman’s H. The most important interaction was between accuracy during in-congruent trials in the *Face Recognition* test and RT in the C*hoice Reaction Time* test, which were present in all models except in the boosted model for cluster D. This pattern was followed by interactions between RT in the *Continuous Performance* test and accuracy during incongruent trials in the *Face Recognition* test as well as RT in the C*hoice Reaction Time* test (present in all models except for random forest models cluster C and D). All other interactions were present in only half or less of the models. Yet, regarding the two different models for PTSD cluster C and D, these were the same within each outcome. Furthermore, there was a unique interaction for predicting PTSD cluster D between accuracy during congruent trials in the *Face Recognition* test and RT in the *Go/No-Go* task This was followed by an interaction between the latter and RT in the *Continuous Performance* test (this was also present in the model for overall PTSD severity). Finally, interactions with time to completion in the D*igit Lette*r test were unique for predicting PTSD cluster C by means of the random forest model, whereas interactions including errors in the *Verbal Interference* test were only occurring in the random forest model for PTSD cluster D.

## Discussion

The aim of the current study was to characterize the prospective nature and predictive accuracy of cognitive processing and emotion recognition on the course of shared and distinct PTSD dimensions by comparing the stability and accuracy of distinct machine learning algorithms. Altogether, decision tree ensemble learners were more effective than single-tree models as well as SVMs and Neural Networks. Further, we found that the variable set was equally predictive of all symptom clusters, accounting for moderate variance across all clusters. High variability in fit indices as indicated by large confidence intervals might have prevented tests from reaching statistical significance.

Sustained attention and emotion recognition bias during incongruent stimuli demonstrated the greatest predictive impact across symptom clusters. In contrast, the domains of working memory/attention and verbal learning, cognitive flexibility and emotion recognition during congruent stimuli were ranked among the least important predictors. Specifically discriminating between PTSD symptom clusters, alterations in inhibitory performance as well as an interaction between emotion recognition and processing speed were visible for symptoms re-experiencing, alterations in cognition and mood and hyper-arousal. Furthermore, for re-experiencing interactions between sustained attention with information processing speed and emotion recognition were unique. Regarding PTSD symptoms of avoidance, there was no involvement of inhibitory processes. However, information processing speed as a main effect was only important in this cluster. Alterations in cognition and mood were furthermore described by interactions between inhibitory performance and processing speed as well as emotion recognition. Finally, for hyper-arousal no other unique pattern was found.

The current findings suggest that alterations in higher-order executive functioning like action inhibition or sustained attention and processing of emotionally incongruent cues on the affective side are relevant for the emergence of PTSD symptoms. This is consistent with empirical findings regarding the role of cognitive and emotional information processing as a mechanism of PTSD development. It is known that that attentional resources are shifted to trauma-related cues after a traumatic event in order to quickly evaluate these stimuli as potentially threatening [9]. Further, co-occurring alterations in inhibitory control lead to an impaired disengagement of attention from these trauma-related cue. Therefore, these processes might result in the evolvement of repeatedly occurring pictures and memories of the traumatic event that are difficult to control.

Symptoms of avoidance might further evolve as a failed coping strategy, whereas altered speed in processing could aid in the identification and subsequent avoidance of potentially threatening situations [9]. Also the notion of impairment in disengagement from trauma-related cues fits well with the interaction between cognitive domains and altered emotion recognition capabilities [8]. Emotion-related cues are known to affect memory capacities and stimuli with high emotional load might be stored differently than more neutral cues [50]. Interacting with cognitive processing, this aspect might further contribute to the evolvement of a trauma-specific memory.

In this study, several of the previously empirically identified cognitive processing and emotion recognition markers were less relevant in the complex interaction models [9, 10]. In addition, not all tests for one specific cognitive domain were relevant. A potential explanation is that cognitive tests do not always map onto a single domain and there is controversy regarding the correct allocation (c.f [10, 13].). Most likely, tests also measure other neuro-cognitive functions not related to PTSD symptoms. Furthermore, differences might also be explained by characteristics of the sample. Finally, these domains may overlap substantially in the variance in predication of PTSD. Whilst in most studies patients with clinically endorsed PTSD symptoms were compared to healthy controls, symptom severity in the current study were low and a dimensional scoring was applied.

Overall, the study confirms the power of data-driven models for merging a large set of diverse information in a useful way. Moreover, it suggests that cognitive processing and emotion recognition characteristics do not fully characterize PTSD symptoms but combining several tests might lead to a substantial better mechanistic understanding of how PTSD symptoms develop.

Including these factors, for example in future neuroimaging studies might have the potential to provide further comprehension of underlying neural circuit dysfunction. However, current results must be confirmed and extended in independent samples to be actionable clinically. With respect to the limited sample size and despite careful guarding against overfitting, it cannot be completely ruled out that the findings are specific to the current sample. It remains to be investigated whether findings generalize to other settings. Moreover, future large-scale investigations should also incorporate diverse f risk factors for PTSD symptoms beyond neuro-cognitive and affective processing (e.g. socio-demographic factors, initial level of posttraumatic stress symptoms, lifetime traumatic load). Furthermore, it is not known whether the model can be applied to clinical samples with more severe PTSD symptoms.

Regarding clinical implications, the current work points to the feasibility of cognitive and emotional tasks to identify risk for PTSD by means of machine-learning models. From a long-term perspective, such approaches can support clinical decision making in order to assess risk for PTSD. This could aid the allocation of early resources for preventing PTSD. In doing so, future research should also focus on predicting PTSD diagnosis instead of continuous PTSD symptoms.

### Limitations

Due to the limited sample size, a-priori theory-based rigor reduction of relevant variables had to be carried out. Furthermore, collinear predictors had to be removed before model building. In addition, we did not explicitly control for traumatic brain injury, and having included these aspects would have expanded the understanding even further. Yet, in comparison to a normative cohort, there was no evidence for severe impairment. In addition, we do not know if cognitive processing has been altered in response to trauma exposure or constitutes a pre-trauma risk factor [15], since there was no pre-assessment. Whilst the current model suggests an underlying causality due to the longitudinal design, it is not an experiment under controlled settings. Accordingly, causality of effects is not conclusive.

Furthermore, the best practice recommendation of randomly splitting the dataset into a training and test set was not feasible because all data was needed for model building. Using a separate test set is recommended in order to avoid that the algorithm “learns” associations between predictor and outcome variables that are specific to the dataset but cannot be generalized. Furthermore, it is unclear how PTSD symptom trajectories of individuals might develop over the course of time after 3 months. Long-term prediction of PTSD symptoms that extends beyond the current time frame is advisable. Finally, there was an under-representation of assaults and an over-representation of bicycle accidents when comparing completers with non-completers.

## Conclusion

The current study demonstrates that data-driven models can contribute to the understanding of PTSD. By including a large number of features, machine learning algorithms can provide new insights in otherwise hidden and complex interconnections between cognitive and emotional information processing. Hereby, the current research points to the importance of simultaneously including measures of higher-order cognitive functioning and emotion recognition in future studies in order to understand underlying alterations related to the development of PTSD symptoms. Future large-scale investigations are needed to validate the current findings and being capable of including a more diverse set of variables with other risk factors.

## Supplementary information

**Additional file 1.**

## Data Availability

The datasets generated and/or analyzed during the current study are not publicly available due to ethical reasons but are available from the corresponding author on reasonable request.

## Abbreviations

CART: Classification and Regression Trees
CP: Continuous Performance
CRT: Choice Reaction Time
DL: Digit-Letter
DSM-5: Diagnostic and Statistical Manual 5th Version
FR I-acc: Face recognition accuracy during incongruent trials
PCL-5: Posttraumatic Symptom Checklist for DSM-5
PTSD: Posttraumatic Stress Disorder
RF: Random Forests
RMSE: Root means squared error
RT: Reaction Time
SVM: Support Vector Machines
VI: Verbal Interference
