# Supplementary material for: Utilization of machine learning to test the impact of cognitive processing and emotion recognition on the development of PTSD following trauma exposure
Source: BMC Psychiatry. 2020 Jun 23;20:325. doi: 10.1186/s12888-020-02728-4 (PMC7310383; doi:10.1186/s12888-020-02728-4)
Supplement: Supplementary file 1 — Additional file 1. [file 12888_2020_2728_MOESM1_ESM.docx]

**Online Supportive Material**

Utilizing machine learning based methods to identify the impact of cognitive processes and emotion recognition on the development of PTSD following trauma exposure

Authors: Mareike Augsburger, Isaac R. Galatzer-Levy

Table 1A

*Z-standardized deviations from the normative cohort using peer regression modeling (PRM) for age, gender, and level of education.*

|  | **Mean** | **SD** |
| --- | --- | --- |
| **Choice Reaction Time** | .03 | 1.89 |
| **Cont. Performance Errors** | -.42 | 2.27 |
| **Cont. Performance Reaction Time** | -1.22 | 1.46 |
| **Go/No-Go Reaction Time** | -.84 | .70 |
| **Go/No-Go Errors** | .08 | .91 |
| **Digit Span Recall** | -.25 | 2.64 |
| **Verbal learning Errors** | .06 | 1.64 |
| **Digit-Letter-test Errors** | -.14 | 1.21 |
| **Digit-Letter-test Completion time** | .02 | 1.87 |
| **Verbal Inference Errors** | .07 | .51 |
| **Face recognition accuracy** | .17 | .95 |

*Note. Positive values indicate above average performance, and negative numbers indicate below average performanc.*

**Description of models**

***Support Vector Machines (SVMs).*** Support Vector Regression with Radial Basis Function Kernel was applied (1). Hereby, predictors are mapped into a high-dimensional feature space. Subsequently, a linear separation (hyperplane) is calculated that aims at minimizing the error. Hereby, a threshold margin (ε) is defined tries to be maximized. In order to avoid overfitting, a cost parameter (C) controls model complexity. It specifies the extent of deviations from ε that is allowed and therefore penalizes too large residuals. As suggested, a grid of 15 values of C between 2^-2^, 2^-1^, …, 2^12^ was searched for the best solution (2).

***Tree-based models Regression Trees.*** In decision trees, the outcome variable is predicted by partitioning each predictor based on a series of if-then statements. These recursive splits are made using the Classification and Regression Tree approach (3): Each value of every predictor in the entire data set is searched for the best split with lowest sums of squared errors of two groups. This process is continued in an iterative process within each group, resulting in a growing tree until a terminal node is reached. To guard against overfitting, model complexity (size of the tree) is reduced by penalizing tree growth with its error rate. Accordingly, full-grown trees are pruned back until the best tradeoff between error rate and tree size is reached, see (3). In the current analysis, 30 complexity parameters were tested.

***Bagged Regression Trees.*** Bootstrap aggregation (bagging) is an extension of models for creating prediction ensembles (see 4). It combines several decision trees created by repeated resampling of the data and averaging resulting trees. This leads to reduced variance, therefore better predictions and also more stable results (2). In the current analysis, CART above-described trees were bagged (4).

***Boosted Regression Trees.*** Stochastic gradient boosting machines (GBM) are another powerful tree-ensemble method. GBM initially start with the prediction of the mean response. In an iterative process, prediction continues based on the calculated residuals that could not explained in the step before. At each step, the algorithm aims at minimizing the squared error. This process continues until the final number of trees is reached. In this analysis, number of trees were chosen between 100-1000 in steps of 50. Additionally, several parameters can be specified in order to increase stability and avoid overfitting: Bag fraction defines a randomly chosen subsample of data taken into account at each iteration. As recommended it was set to .5 (5). Tree depth is the interaction depth (an interaction depth of 2 can be thought as two-way interaction) and was tuned between 1-4. . Additionally, minimal observations at each terminal node were varied between 2,3, or 4. Finally, the shrinkage describes the fraction of the predicted value at each step that is added to the overall model. This model was tested with a shrinkage of both .01 and .1 following suggestions of Kuhn and Johnson (2). See

***Random Forests (RF).*** RF (6) is also a tree-based ensemble learning technique. In contrast to bagged trees, RF do not include the entire set of predictors at each step of the tree-building procedure but a random subset at each iteration. Like in bagged trees, predictions are averaged. Including this random component of a subset of predictors leads to less correlated trees and therefore better prediction (2). In the current analysis, the number of predictors was a tuning grid of ten values ranging from 2-12. In total, 1000 trees were grown, following the recommendations of Kuhn and Johnson (2). For further details see (6).

***Neural Networks***.  A feed-forward network was applied (7). This algorithm combines linear combinations of predictors into multiple hidden units. There is no restriction about the number of predictors modeling a hidden unit. In a second step, the outcome is modelled by linear combinations of hidden units, aiming at minimizing the sum squared residuals. To increase stability, five random starting values were set and averaged accordingly. In order to prevent overfitting, neural networks are regularized by a weight decay (λ) that penalizes large coefficients without significant error reduction. A grid of λ between .1, .01, and .001 was searched. Number of hidden units was set between 1 and 27 in steps of two.

(47)

Table 2A

*Final SVM tuning parameters and mean fit indices for the prediction of PTSD total symptom severity and PTSD clusters*

|  | **C** | **RMSE** | **R-squared** |
| --- | --- | --- | --- |
| **PTSD total** | 0.5 | 15.35 | 0.16 |
| **PTSD B** | 1 | 3.87 | 0.25 |
| **PTSD C** | 1 | 3.87 | 0.25 |
| **PTSD D** | 0.5 | 6.08 | 0.16 |
| **PTSD E** | 0.55 | 4.99 | 0.12 |

*Note*. C = Complexity parameter. RMSE = Root mean squared error. Sigma was constant at a value of .14.

Table 3A

*Final CART tuning parameters and mean fit indices of CART and bagged models for the prediction of PTSD total symptom severity and PTSD clusters*

|  | **cp** | **RMSE**  **CART** | **R-squared**  **CART** | **RMSE**  **bagged** | **R-squared**  **bagged** |
| --- | --- | --- | --- | --- | --- |
| **PTSD total** | 0.04 | 15.53 | 0.15 | 14.32 | 0.19 |
| **PTSD B** | 0.09 | 3.88 | 0.27 | 3.58 | 0.35 |
| **PTSD C** | 0.13 | 2.03 | 0.16 | 1.96 | 0.25 |
| **PTSD D** | 0.04 | 6.46 | 0.15 | 5.88 | 0.21 |
| **PTSD E** | 0.16 | 4.64 | 0.26 | 4.76 | 0.19 |

*Note*. cp = cost-complexity parameter. RMSE = Root mean squared error.

Table 4A

*Final Boosted trees tuning parameters and mean fit indices for the prediction of PTSD total symptom severity and PTSD clusters*

|  | **S.** | **Tree depth** | **Min. obs.** | **# trees** | **RMSE** | **R-squared** |
| --- | --- | --- | --- | --- | --- | --- |
| **PTSD total** | 0.01 | 1 | 4 | 150 | 14.21 | 0.28 |
| **PTSD B** | 0.01 | 2 | 4 | 150 | 3.53 | 0.35 |
| **PTSD C** | 0.01 | 3 | 3 | 100 | 1.96 | 0.22 |
| **PTSD D** | 0.01 | 3 | 3 | 150 | 5.81 | 0.2 |
| **PTSD E** | 0.01 | 1 | 3 | 150 | 4.62 | 0.23 |

*Note*. S. = shrinkage, min. obs. = minimum number of observations per node, # trees = number of trees, RMSE = Root mean squared error.

Table 5A

*Final Random forest tuning parameters and mean fit indices for the prediction of PTSD total symptom severity and PTSD clusters*

|  | **mtry** | **RMSE** | **Rsquared** |
| --- | --- | --- | --- |
| **PTSD total** | 2 | 14.22 | .28 |
| **PTSD B** | 3 | 3.57 | .33 |
| **PTSD C** | 4 | 1.92 | .25 |
| **PTSD D** | 4 | 5.82 | .20 |
| **PTSD E** | 2 | 4.75 | .17 |

*Note*. Mtry = number of predictors, RMSE = Root mean squared error. Sigma was constant at a value of .21.

Table 6A

*Neural network tuning parameters and mean fit indices for the prediction of PTSD total symptom severity and PTSD clusters*

|  | **λ** | **# hidden units** | **RMSE** | **R-squared** |
| --- | --- | --- | --- | --- |
| **PTSD total** | 0 | 1 | 15.29 | 0.25 |
| **PTSD B** | 0 | 1 | 3.82 | 0.28 |
| **PTSD C** | 0.1 | 1 | 2.11 | 0.14 |
| **PTSD D** | 0.01 | 1 | 6.63 | 0.12 |
| **PTSD E** | 0.1 | 1 | 5.1 | 0.14 |

*Note*. RMSE = Root mean squared error.

**
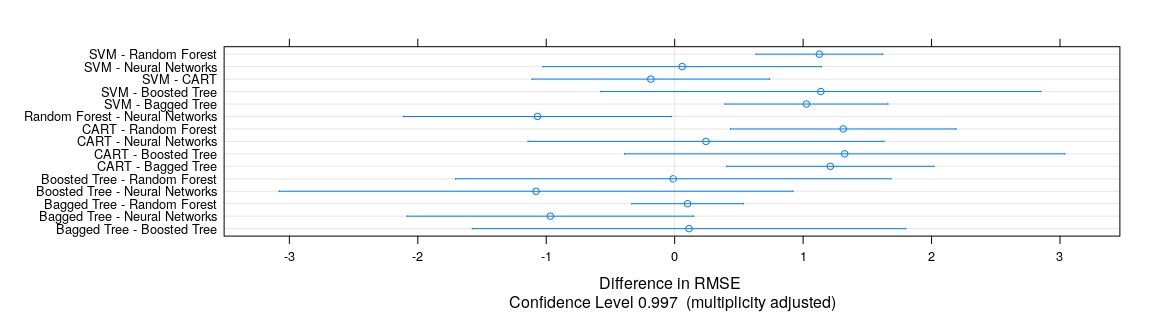
**

Figure S1. Differences in RMSE for prediction models of overall PTSD symptom severity.


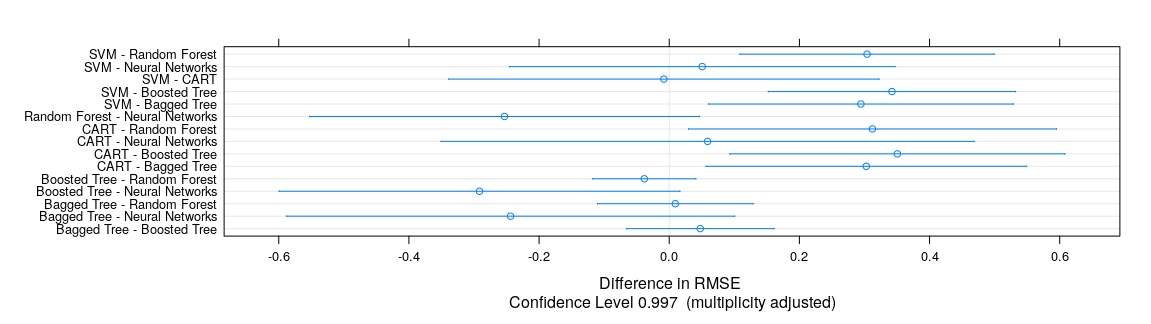
Figure S2. Differences in RMSE for prediction models of PTSD cluster B.


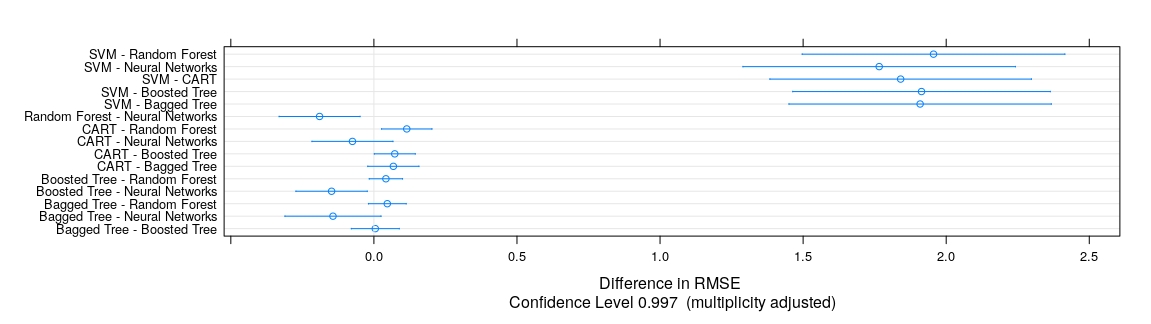
Figure S3. Differences in RMSE for prediction models of PTSD cluster C.


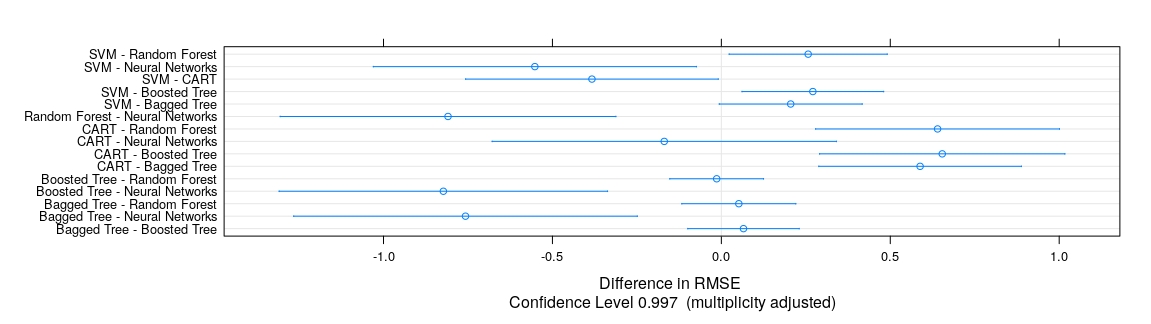


Figure S4. Differences in RMSE for prediction models of PTSD cluster D.


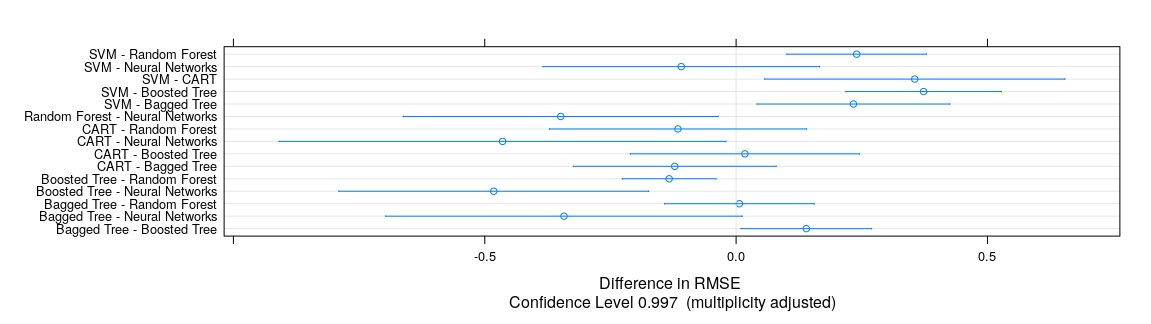
Figure S5. Differences in RMSE for prediction models of PTSD cluster E.

**References**

1. Drucker H, Burges CJC, Kaufman L, Smola A, Vapnik V. Support Vector Regression Machines. In: Mozer MC, Jordan JI, Petsche T, editors. Neural Information Processing Systems. MIT Press; 1997. p. 155–61.

2. Kuhn M, Johnson K. Applied predictive modeling. Corrected 5th printing. New York: Springer; 2016. 600 p.

3. Breiman L, Friedman JH, Stone CJ, Olshen RA, editors. Classification and regression trees. Repr. New York: Chapman & Hall; 1984. 358 p.

4. Breiman L. Bagging predictors. Mach Learn. 1996 Aug;24(2):123–40.

5. Friedman JH. Greedy Function Approximation: A Gradient Boosting Machine. Ann Stat. 2001;29(5):1189–232.

6. Breiman L. Random Forests. Mach Learn. 2001;5–32.

7. Titterington M. Neural networks: Neural networks. Wiley Interdiscip Rev Comput Stat. 2010 Jan;2(1):1–8.
